# The effect of occupational coping self-efficacy on presenteeism among ICU nurses in Chinese public hospitals: a cross-sectional study

**DOI:** 10.3389/fpsyg.2024.1347249

**Published:** 2024-01-31

**Authors:** Jijun Wu, Yuxin Li, Qin Lin, Jiquan Zhang, Zhenfan Liu, Xiaoli Liu, Xian Rong, Xiaoli Zhong

**Affiliations:** ^1^Department of Cardiology, Deyang People’s Hospital, Deyang, China; ^2^School of Nursing, North Sichuan Medical College, Sichuan, China; ^3^Shulan International Medical College, Zhejiang Shuren University, Hangzhou, China; ^4^Department of Nursing, Deyang People’s Hospital, Deyang, China; ^5^Sichuan Nursing Vocational College, Sichuan, China

**Keywords:** ICU nurses, presenteeism, coping self-efficacy, nursing management, influencing factors, China

## Abstract

**Background:**

Nurses are the largest occupational group in the health field, with inestimable value in realizing universal health coverage, and nurses’ physical and mental health has become an ordinary global reality. Compared with explicit absence, nurses’ presenteeism has a more lasting impact and significant harm and loss. It has become an essential factor affecting nurses’ physical and mental health, declining quality of healthcare services, and elevated healthcare-related risks. There is a lack of research exploring whether occupational coping self-efficacy influences nurses’ presenteeism behavior, especially in less-developed regions of China.

**Objective:**

This study aimed to investigate the current status of ICU nurses’ occupational coping self-efficacy and presenteeism in public hospitals in western China and to explore the impact of ICU nurses’ occupational coping self-efficacy on presenteeism.

**Methods:**

A cross-sectional research design selected 722 ICU nurses in western China from January to February 2023 as survey respondents. A general information questionnaire, Occupational Coping Self-Efficacy Scale (OCSE-N), and Stanford Presenteeism Scale (SPS-6) were used. SPSS 21.0 software was used for statistical analysis. Pearson correlation analysis and multivariate hierarchical regression were used to explore the influence of ICU nurses’ occupational coping self-efficacy on presenteeism.

**Results:**

A total of 722 ICU nurses completed the questionnaire. The OCSE-N score of ICU nurses was (22.24 ± 6.15), and the SPS-6 score was (16.83 ± 4.24). The high presenteeism was 67.23%. Correlation analysis showed that in ICU nurses, OCSE-N total score was negatively correlated with SPS-6 total score (*r* = −0.421, *p* < 0.05), indicating that the higher the level of occupational coping self-efficacy, the lower the presenteeism. Multiple hierarchical regression analysis showed that occupational coping self-efficacy strongly predicted presenteeism, accounting for approximately 18.35% of the total variance.

**Conclusion:**

There is a correlation between ICU nurses’ occupational coping self-efficacy and presenteeism, and nurses’ occupational coping self-efficacy affects presenteeism differently. Managers should pay attention to nurses’ occupational coping self-efficacy to promote nurses’ presenteeism reduction.

## 1 Introduction

Presenteeism, also known as impaired health productivity, refers to an employee who is present at work but cannot effectively engage in their job due to physiological or psychological factors, manifesting in decreased productivity and reduced work engagement (Turpin et al., 2004). Presenteeism significantly affects the physical and mental health of employees and patient safety. The impact of presenteeism is more long-lasting, harmful, and costly than visible absences. Research has shown that presenteeism exists in all sectors of the population but is particularly pronounced in the healthcare sector, where it is approximately four times more common in nurses than in other sectors (Kim et al., 2019; Simonetti et al., 2021). Previous studies have shown that the presenteeism rate of Spanish nurses is about 30% (Freeling et al., 2020), Portuguese nurses’ presenteeism rate is 55% (Mosteiro-Díaz et al., 2020), Finnish registered nurses’ presenteeism rate is about 37.4% (Rantanen and Tuominen, 2011), U.S. nurses’ presenteeism rate is 52.65% (Warren et al., 2011), and China’s presenteeism rate of nurses is as high as 70.6% (Li et al., 2021). Studies have shown that presenteeism in Australia costs the economy approximately $34.1 billion annually, while presenteeism in the U.S. nursing industry is roughly $12 billion annually (Letvak et al., 2012). Various factors are associated with presenteeism among nurses, such as social work environment, occupational stress, shortage of human resources, and health conditions differences in the system of health service provision between countries (Santos et al., 2021). Nurses’ presenteeism not only affects nurses’ physical and mental health but also leads to a multiplication of nursing errors (e.g., patient falls and medication administration errors), which inadvertently increases the burden of patient care and causes severe damage to the quality and image of nursing services (Letvak et al., 2012; Lui et al., 2018). Consequently, presenteeism has become a significant public health issue globally today. It has attracted the attention of multidisciplinary researchers in organizational psychology, health psychology, and management in recent years. Therefore, given the impact of presenteeism on nurses’ health and quality of care, there is a need to explore the implications of this phenomenon further.

By the end of 2022, China’s total number of registered nurses exceeded 5.2 million, with 3.56 registered nurses per 1,000 population, lower than the global level of 3.69 nurses per 1,000 population in 2018, according to the report (Liu et al., 2020). The intensive care unit (ICU), as a key and specialized area for life support of critically ill patients in medical institutions, requires sufficient human resources, necessary technology, and highly sophisticated equipment for monitoring and treatment of critically ill patients and is a high-load, high-pressure and high-risk clinical department in medical institutions (Rewa et al., 2018). The presenteeism of ICU nurses is also exceptionally high due to reasons such as dedication, fear of coworker overload, performance bonuses, and difficulty in getting another professional to take over.

Occupational coping self-efficacy refers to nurses’ subjective evaluations and perceptions of their ability to cope effectively and complete nursing care, including nurses’ sense of confidence in their work goals and their sense of judgment about their ability to act and handle their behavior (Pisanti et al., 2008). Occupational coping self-efficacy consists of 2 dimensions: occupational burden (effectively coping with work tasks) and relationship difficulties (effectively dealing with interpersonal relationships between healthcare professionals and patients). As an essential internal resource, occupational coping self-efficacy plays a vital role in developing an individual’s occupational health. Research has shown that occupational coping self-efficacy is essential for nurses to establish good interpersonal relationships, cope with work stress, and perform their job duties with positive beliefs (Jex et al., 2001; Shahrour and Dardas, 2020). Theoretically, occupational coping self-efficacy may predict the occurrence of presenteeism through an individual’s self-efficacy to effectively cope with work tasks and good interpersonal relationships.

Research suggests that occupational coping self-efficacy enhances nurses’ psychological well-being, reduces burnout, and improves work engagement (Laschinger et al., 2015; Pisanti et al., 2015; Shoji et al., 2016). Several studies have noted that presenteeism is particularly prominent among nurses in low-income or underdeveloped areas and can lead to negative work-related consequences (Galon et al., 2014). In addition, low levels of occupational coping self-efficacy were associated with higher levels of presenteeism, either directly or indirectly, by moderating work stress and better adapting and dealing with work, thus reducing the incidence of presenteeism. Self-development theory suggests that self-development stops when the subject cannot adapt to environmental changes and severe job shocks, hindering actively seeking feedback (McLean et al., 2007). Occupational coping self-efficacy refers to effectively responding to work tasks and dealing with interpersonal relationships. It requires necessary skills, indicating the employee’s self-confidence in accomplishing work tasks. The lack of coping self-efficacy may be an essential factor that leads to the inability to engage in the work effectively, so the employee feels inefficient.

Few studies have investigated the relationship between ICU nurses’ occupational coping self-efficacy and presenteeism, and there needs to be research on the relationship between ICU nurses’ occupational coping self-efficacy and presenteeism in Chinese public hospitals. For these reasons, conducting this study based on self-development theory is essential, especially in developing countries where human resources are in short supply. In addition, we investigated the influential factors associated with presenteeism. In traditional Chinese Confucian values, the subject of work is, first and foremost, self-development, which positively affects the quality of work. Although nurses are subjectively severe about their work, they are influenced by their self-coping skills, which leads to unconscious influences on the level of work engagement, thus generating presenteeism.

Therefore, this study aimed to determine the relationship between occupational coping self-efficacy and presenteeism among Chinese ICU nurses and to provide a reference for nursing managers to develop targeted interventions to reduce presenteeism among nurses. Based on previous research (Fiorini et al., 2022; Liu et al., 2023), this study proposed the following hypotheses: (1) ICU nurses have high levels of presenteeism, which is influenced by multiple factors such as age, length of service, and health status; (2) occupational coping self-efficacy is negatively related to presenteeism and significantly predicts presenteeism.

## 2 Methods

### 2.1 Participants

This study was a cross-sectional research design with a convenience sampling method conducted from January to February 2023 in western China. Finally, 722 ICU nurses were included. The sample size calculation formula (Ni et al., 2010), set = 0.05, according to the pre-survey results ICU nurses’ presenteeism total score standard deviation = 4.24; it is hoped that the allowable error is not more than 2, the control value of this study was set to 0.5, resulting in = 276, considering 20% of invalid questionnaires, the sample size of about 331. Inclusion criteria: obtaining the vocational qualification certificate for nurses, working in an ICU clinic for more than one year, knowing the purpose and significance of this study, and voluntarily participating in this study. Exclusion criteria: internship, training, advanced training nurses; absent nurses on maternity leave, personal leave, sick leave, etc. A total of 802 questionnaires were recovered, excluding questionnaires with apparent regularity (the same answers to 6 or more consecutive entries) or questionnaires with inconsistent logic (e.g., inconsistency between length of service and title), a total of 722 valid questionnaires were recovered, and the recovery rate of valuable questionnaires was 90.02%.

### 2.2 Procedures

All the data in this study were collected through the electronic questionnaire “Questionnaire Star.” With the support of the director of the nursing department of each hospital, an ICU head nurse was identified as the survey liaison for this study. The ICU head nurse sent the link to the electronic questionnaire to the ICU nurses’ WeChat work group. The first page of the questionnaire explained the purpose, significance, method, and precautions for filling in the questionnaire. The first page of the questionnaire explained the importance of filling out the survey. To ensure the reliability of the questionnaire data, the entries were set as mandatory options, and each cell phone was limited to fill in and submit once.

### 2.3 Measures

A self-administered questionnaire was used to collect basic information about ICU nurses, including gender, age, marital and childbearing status, education, title, position, years of experience in ICU, average monthly income, type of contract, self-assessed physical health, presence of chronic illnesses, allocation of human resources in the ICU, presence of on-call duty, and whether they had suffered from workplace violence in the past year.

The Chinese version of the Occupational Coping Self-Efficacy Scale for Nurses (OCSE-N) (Zhai et al., 2021), which has the same structure as the Occupational Coping Self-Efficacy Scale developed by Pisanti et al. (2008), was used. The scale consists of 9 items, including two dimensions of individual occupational burden and relationship difficulties. It measures how confident nurses are in coping quickly with stressful scenarios often encountered in the workplace. The scale is scored on a 5-point Likert scale, with the total score ranging from 9 to 45, and the higher the total score, the higher the level of occupational coping self-efficacy. The Cronbach’s alpha coefficients of the English version of the original scale were 0.77 and 0.79 for occupational burden and relationship difficulties, respectively, and the Cronbach’s alpha of the Chinese version of the Nurses’ Occupational Coping Self-Efficacy Scale was 0.882, with a retest reliability of 0.991.

The Chinese version of the Stanford Presenteeism Scale-6 (SPS-6) was used to measure the individual’s presenteeism in the past month (Zhao et al., 2010), and the structure of the scale was basically the same as that of the Stanford Presenteeism Scale developed by KOOPMAN (Koopman et al., 2002). The scale consists of six items, mainly measuring the nurses’ work energy and work limitations, of which item 5, “In the past month, I was able to concentrate on my work despite health problems” and item 6, “In the past month, I felt that I had enough energy to do all my work despite health problems” and “In the past month, I felt that I had enough energy to do all my work despite health problems.” I feel energized and can do all my work” were reverse scored. A 5-point Likert scale was used, with a total score range of 6 to 30, with higher scores indicating more severe presenteeism. The median SPS-6 score was used as the cutoff for low and high presenteeism. The Cronbach’s alpha coefficient for the English version of the scale was 0.80, and that for the Chinese version was 0.76.

### 2.4 Statistical analysis

Statistical analyses were performed using SPSS 21.0 software, and the test level was set at α = 0.05. Categorical descriptions of general information characteristics were expressed as *n* (%), levels of presenteeism between categories were expressed as mean ± standard deviation, and comparisons between groups were made using independent samples t-tests or one-way ANOVA tests. Pearson correlation analysis explored the correlation between occupational coping self-efficacy and presenteeism. Multivariate hierarchical regression analyses were conducted with presenteeism as the dependent variable, demographic variables as the control variables, and occupational coping self-efficacy as the independent variable to test occupational coping self-efficacy as a predictor of the impact of presenteeism scores.

### 2.5 Ethical considerations

The Ethics Committee of the People’s Hospital of Deyang City (2021-04-056-K01) reviewed and approved this study, and the subjects participated voluntarily. Our study adheres to the Helsinki Declaration by the WMA.

## 3 Results

### 3.1 Relationship between general information characteristics of ICU nurses and presenteeism

Table 1 shows the general characteristics of the 722 ICU nurses. Predominantly female (90.72%); less than 40 years of age (91.55%); married (69.62%); bachelor’s degree (82.96%); nurse practitioner (48.06%); clinical nurse (79.22%); more than 10 years of ICU experience (42.66%); average monthly income less than 8,000 yuan (67.73%); and labor contract (79.50%); Good physical condition (58.31%); chronic disease (14.54%); high level of perceived work stress (46.68%); ICU human resource allocation less than 1:2.5–3 (59.56%); night shift rotation (84.35%); and suffering from workplace violence in the past year (33.24%).

**Table 1:** Presenteeism according to the general characteristics of ICU nurses (*n* = 722).

Variables		*n* (%)	Presenteeism (mean ± SD)	*t* or *F*	*p*-value
Gender	Male	67 (9.28)	15.22 ± 4.88	1.024	0.306
	Female	655 (90.72)	15.80 ± 4.33		
Age	<30	292 (40.44)	15.81 ± 4.12	2.200	0.087
	30 ~ <40	369 (51.11)	15.91 ± 4.60		
	40<50	52 (7.20)	14.27 ± 3.89		
	≥50	9 (1.25)	15.33 ± 5.34		
Marital or childbearing status	Unmarried	205 (28.39)	16.28 ± 4.18	2.252	0.081
	Married with no children	84 (11.63)	16.19 ± 3.71		
	Married with children	418 (57.89)	15.39 ± 4.59		
	Divorced/widowed	15 (2.08)	16.00 ± 4.09		
Highest education	Junior college and below	113 (15.65)	15.59 ± 4.34	0.387	0.679
	Undergraduate	599 (82.96)	15.79 ± 4.40		
	Master degree or above	10 (1.39)	14.70 ± 4.14		
Professional title	Nurse	111 (15.37)	15.93 ± 4.35	3.369	0.018
	Nurse practitioner	347 (48.06)	16.10 ± 4.15		
	Nurse supervisor	236 (32.69)	15.37 ± 4.69		
	Deputy chief nurse and above	28 (3.88)	13.75 ± 4.20		
Position	Clinical nurse	572 (79.22)	15.96 ± 4.31	3.544	0.029
	Nursing team leader	94 (13.02)	15.11 ± 4.96		
	Head nurse	56 (7.76)	14.63 ± 3.91		
Years of ICU work	1 ~ <5	277 (38.37)	15.47 ± 4.15	2.951	0.053
	5 ~ <10	237 (32.83)	16.31 ± 4.24		
	≥10	308 (42.66)	15.47 ± 4.79		
Income per month	1 ~ <6,000	173 (23.96)	15.98 ± 4.38	0.959	0.412
	6,000 ~ <8,000	316 (43.77)	15.91 ± 4.40		
	8,000 ~ <10,000	171 (23.68)	15.31 ± 4.29		
	≥10,000	62 (8.59)	15.47 ± 4.57		
Type of contract	Staffing of government-affiliated institutions	148 (20.50)	15.03 ± 4.07	2.223	0.026
	Labor contract	574 (79.50)	15.93 ± 4.45		
Physical health condition	Good	421 (58.31)	14.59 ± 4.25	50.459	<0.001
	General	269 (37.26)	16.99 ± 3.92		
	Worse	32(4.43)	20.53 ± 3.90		
Suffering from chronic disease	Yes	105 (14.54)	17.08 ± 4.14	3.383	0.001
	No	617 (85.46)	15.22 ± 4.39		
Perceived work stress	Low	16 (2.22)	11.44 ± 4.05	36.236	<0.001
	Moderate	369 (51.11)	14.73 ± 3.96		
	High	337 (46.68)	17.07 ± 4.43		
ICU human resource allocation	<1:2.5 ~ 3	430 (59.56)	15.45 ± 4.53	3.754	0.024
	=1:2.5 ~ 3	178 (24.65)	15.87 ± 3.98		
	>1:2.5 ~ 3	114 (15.79)	16.69 ± 4.33		
Night shift	Yes	609 (84.35)	16.01 ± 4.29	3.852	<0.001
	No	113 (15.65)	14.30 ± 4.61		
Experienced workplace violence in the past year	Yes	240 (33.24)	17.29 ± 3.96	6.872	<0.001
	No	482 (66.76)	14.98 ± 4.39		

### 3.2 Descriptive statistics and correlation matrix among key general characteristics, occupational coping self-efficacy, presenteeism of ICU nurses

Table 1 shows the results of descriptive statistics of ICU nurses’ occupational coping self-efficacy and presenteeism. The results of this survey showed that 722 ICU nurses had an occupational coping self-efficacy score of 27.32 ± 3.00 (median score of 22.5) and a presenteeism score of 15.75 ± 4.39 (median score of 15), with a median cutoff of 16 on the SPS-6 total score, and a high presenteeism of 56.4%.

The correlations between the main general characteristics of ICU nurses (with significant differences in presenteeism in Table 1), occupational coping self-efficacy, and presenteeism are shown in Table 2, where title, position, contract type, self-rated physical health, perceived work stress, ICU human resource allocation, and night shift rotation were positively correlated with latent absenteeism, and having a chronic illness, and having been exposed to workplace violence in the past year were negatively associated with latent absenteeism. Self-rated physical health, perceived work stress, and occupational coping self-efficacy were negatively correlated; having a chronic illness and experiencing workplace violence in the past year were positively correlated; and occupational coping self-efficacy was negatively correlated with presenteeism.

**Table 2:** Descriptive statistics of nurses’ occupational coping self-efficacy, presenteeism, and correlation matrix with nurses’ key general characteristics.

Variables	Mean ± *SD*	1 (*r*_s_)	2 (*r*_s_)	3 (*r*_s_)	4 (*r*_s_)	5 (*r*_s_)	6 (*r*_s_)	7 (*r*_s_)	8 (*r*_s_)	9 (*r*_s_)	10 (*r*)	11
1. Professional title	—	1	—	—	—	—	—	—	—	—	—	—
2. Position	—	—	1	—	—	—	—	—	—	—	—	—
3 Type of contract	—	—	—	1	—	—	—	—	—	—	—	—
4. Physical health condition	—	—	—	—	1	—	—	—	—	—	—	—
5. Suffering from chronic disease	—	—	—	—	—	1	—	—	—	—	—	—
6. Perceived work stress	—	—	—	—	—	—	1	—	—	—	—	—
7. ICU human resource allocation	—	—	—	—	—	—	—	1	—	—	—	—
8. Night shift	—	—	—	—	—	—	—	—	1	—	—	—
9. Experienced workplace violence in the past year	—	—	—	—	—	—	—	—	—	1	—	—
10. Occupational coping self-efficacy	27.32 ± 3.00	0.045	0.048	−0.014	−0.220^**^	0.106^**^	−0.215^**^	−0.034	0.045	0.168^**^	1	—
11. Presenteeis-m	15.75 ± 4.39	0.091^*^	−0.088^*^	0.091^*^	0.330^**^	−0.123^**^	0.308^**^	0.101^**^	−0.141^**^	−0.242^**^	−0.223^**^	1

*r* = by Pearson correlation analysis, rs = by Spearman correlation analysis.

*p < 0.05 correlation is significant at the 0.05 level (2-tailed).

**p < 0.01 correlation is significant at the 0.05 level (2-tailed).

### 3.3 Multiple stratified regression analysis: the effect of occupational coping self-efficacy on presenteeism

Stratified regression analysis was used to explore the factors influencing presenteeism among ICU nurses. Stratified regression analyses were conducted with presenteeism as the dependent variable, one-way analysis of statistically significant ICU nurses’ main general characteristics (title, position, contract type, self-rated physical health, having a chronic disease, perceived work stress, ICU human resource allocation, night shift rotation, and exposure to workplace violence in the past year), and occupational coping self-efficacy as the independent variables. The independent variables were included in 2 steps. The main general characteristics significantly different from presenteeism were included as control variables in the first stratum. Career coping self-efficacy was a test predictor variable in the second level. To test for multicollinearity between the variables, multicollinearity analysis was used at each step, and the results showed that the tolerance between the independent variables was more significant than 0.1 and the variance inflation factors were all less than 3. The results showed that there was no severe multicollinearity between the independent variables.

Table 3 Results of stratified regression analyses, Model 1 showed significant differences (*F* = 20.818, *p* < 0.001) in self-rated health (*β* = 1.875, *p* < 0.001), perceived job stress (*β* = 1.700, *p* < 0.001), and exposure to workplace violence in the past year (*β* = −1.469, *p* < 0.001), which accounted for 19.8%.

**Table 3:** Multiple stratified regression analysis of factors influencing presenteeism of ICU nurses.

Variables	Model 1			Model 2		
	*β*	*t*	*p*-value	*β*	*t*	*p*-value
Control variables						
1. Physical health condition	1.875	6.765	<0.001	1.317	5.009	<0.001
2. Perceived work stress	1.700	5.992	<0.001	1.044	3.848	<0.001
3. Experienced workplace violence in the past year	−1.469	−4.536	<0.001	−0.977	−3.211	<0.001
4. Occupational coping self-efficacy						
5. Professional burden	—	—	—	−0.097	−2.047	0.041
6. Difficulty getting along in relationships	—	—	—	−0.484	−6.175	0.000
*F* (*p*-value)		20.818 (<0.001)			30.316 (<0.001)	
*R* ^2^		0.208			0.320	
*R*^2^ change		0.198			0.309	

Dependent variable: presenteeism; *p*-value was derived using multiple regression analysis, α = 0.05. Assignments: self-rated health: good = 1, general = 2, worse = 3; perceived work stress: low = 1, moderate = 2, high = 3; whether or not suffered from workplace violence in the past year: yes = 1, no = 2; actual values of occupational coping self-efficacy substituted.

In Model 2, the vocational coping self-efficacy variable was added, and the study results showed that vocational coping self-efficacy was a significant predictor of hidden absenteeism among ICU nurses, independently explaining 11.1% of presenteeism. Among the occupational coping self-efficacy dimensional variables, difficulty getting along in relationships was the most significant influence (*β* = −0.484, *p* < 0.001), followed by occupational burden (*β* = −0.097, *p* < 0.001).

## 4 Discussion

Overall, ICU nurses had a presenteeism score of 15.75 ± 4.39, with 56.4% high presenteeism, which was higher than the overall estimate of presenteeism detection rate of 49.2%(Min et al., 2022) with the global nursing workforce and was similar to that of Chinese nurses during the pandemic of neo coronavirus pneumonia (15.05 ± 4.52) (Li et al., 2021) but lower than that of nurses in the United Kingdom (17.50 ± 4.22) (Fiorini et al., 2022), higher than that of nurses in Saudi Arabia (21.0 ± 4.3) (Shdaifat, 2023). The reasons for this were analyzed as follows: on the one hand, ICUs are under closed management, and nurses are responsible not only for monitoring and treating patients’ lives but also for various kinds of life care, which makes their workload heavy; on the other hand, the conditions of critically ill patients are changing rapidly, which requires nurses to be on their toes at all times and results in high psychological pressure and occupational tension among nurses, and studies such as those of Banks et al. have also shown that an increase in work-related stress is an independent factor in presenteeism from work. Banks et al. also showed that increased work-related stress is an independent factor influencing presenteeism, and the higher the stress, the higher the rate of presenteeism of nurses (Banks and Pearson, 2021). Secondly, most ICUs in China have a group system of shift work, with a relatively fixed number of staff on each shift and a reasonable mix, and out of professional responsibility, nurses often choose to stick to their jobs when ill health occurs.

In this study, there was a significant relationship between self-assessed health status and presenteeism, with nurses with poorer perceived health status having higher levels of presenteeism, consistent with previous related studies (Jia et al., 2022; Jones et al., 2022). Presenteeism is usually a decrease in productivity caused by illness or extended working hours; therefore, research also usually focuses on the interaction of health and disease, with employees with poorer health status reporting increased levels of hidden presenteeism. The study by Whysall et al. also revealed that chronic non-communicable diseases (e.g., musculoskeletal disorders and psychiatric disorders such as common anxiety, depression, and stress) are the primary health problems that cause presenteeism (Whysall et al., 2018).

In this study, perceived job stress was one factor affecting ICU nurses’ presenteeism; the higher the level of perceived job stress, the higher the presenteeism. Previous studies have shown that nurses’ work stress may lead to sleep disorders, anxiety, and depression and have different degrees of impact on nurses’ work quality, work efficiency, and motivation, which is similar to the results of the present study (Umann et al., 2014; Cicolini et al., 2016). ICU nurses, as the primary caregivers of patients, have far more work pressure, work intensity, workload level, and work hours than medical personnel in other positions, and under the high power of work pressure, medical personnel’s physiological and psychological health is affected. Under high work pressure, medical personnel’s physical and mental health are easily influenced, resulting in presenteeism (van Mol et al., 2018; Ramírez-Elvira et al., 2021).

In this study, having suffered workplace violence in the past year was a risk factor for presenteeism among ICU nurses. Due to the particular object of hospital services, nurses are prone to physical, verbal, and psychological violence from the outside world in the course of their work, which has a different degree of impact on the sense of professional value, professional identity, and willingness to stay in the job (Pol et al., 2019; Yi and Feng, 2022). The reasons for this may be ICU nurses are in the most direct contact with patients and their families in the work process, are the communication bridge between doctors and patients, and may face the risk of being verbally abused and physically violent when communication is poor; secondly, critically ill patients are a high incidence of delirium due to the treatment they receive in closed environments, and this may also cause suffering from workplace violence. All of these workplace violence can be detrimental to nurses’ physical and mental health, occupational fatigue and occupational stress occur, and lead to the occurrence of presenteeism (Conway et al., 2016; Lee and Lee, 2022).

In this study, occupational coping self-efficacy was negatively correlated with presenteeism (*r* = −0.223, *p* < 0.05), indicating that the lower the occupational coping self-efficacy, the higher the probability of nurses experiencing presenteeism. In this case, we conducted a multivariate hierarchical regression analysis to determine how much occupational coping self-efficacy influences presenteeism. As shown in Table 3, in Model 1, self-assessed health status, perceived job stress, and exposure to workplace violence in the past year significantly influenced presenteeism (*F* = 20.818, *p* < 0.001), contributing approximately 19.8% of the total degree of variation in presenteeism, suggesting that self-assessed health status, perceived job stress, and exposure to workplace violence in the past year have limited influence on presenteeism. In Model 2, the results showed that the vocational coping self-efficacy variable significantly influenced presenteeism (*F* = 30.316, *p* < 0.001), contributing about 30.9% of the total variance in hidden absenteeism. This indicates that occupational coping self-efficacy is a significant predictor of presenteeism, consistent with Hypothesis 2. This result is consistent with the results of previous studies in China (Liu et al., 2023). The results of this study indicate that difficulties in getting along in relationships are the strongest predictor of presenteeism. At the same time, the occupational burden has a weaker effect on presenteeism, which may be related to Chinese interpersonal social relationships, which are, in a sense, stable and occupy an essential position in practical activities. Good interpersonal relationships can gain more external support (Jiang et al., 2020; Song and Zhao, 2022). In addition, occupational coping self-efficacy also enhances nurses’ confidence in managing work tasks and motivates nurses to take positive coping measures to deal with dilemmas and overcome difficulties encountered at work. The underlying reasons for the results of this study may be related to the overall low level of education and poor coping ability of Chinese nurses (Fu et al., 2022); their younger age and less available external support (Hao et al., 2022); the shortage of human resources and heavy workload of nurses (Zhang W. Q. et al., 2023); and their poor physical health and prominent occupational-related health problems (Zhang H. et al., 2023).

Finally, we make suggestions to the decision-making departments based on the research results. First, the education department should continue to vigorously implement nursing education to train more nursing talents, alleviate the shortage of nurses’ human resources, and further improve the overall academic level and professional ability of nurses. Secondly, according to the performance of nurses and combined with their titles and years of experience, different welfare benefits should be given to them to form a competitive working atmosphere to stimulate nurses’ motivation to work and reduce the presenteeism of “work without effort”; thirdly, we should pay attention to nurses’ physical and mental health, and reduce the workload or give them rest and humanistic care promptly when they are unwell; fourthly, the education department should continue to implement nursing education to cultivate more nursing talents to alleviate the shortage of nurses’ human resources. Thirdly, pay attention to nurses’ physical and mental health, reduce workload or give rest when nurses are not feeling well, and provide humanistic care. Fourthly, organize regular activities to contact each other and establish good interpersonal relationships to improve nurses’ external support conditions. Fifth, nursing administrators should implement scientific and reasonable scheduling to rationally allocate nurses according to their workload, workload, and work capacity to avoid overloading nurses. Finally, hospitals should provide a safe and comfortable working environment, pay attention to the problem of disproportionate nurse–patient ratio in the department, adjust the bed-nurse balance according to the workload, reduce nurses’ workload and occupational pressure, improve nurses’ job satisfaction and reduce presenteeism. At the same time, we have some suggestions for nurses. First, nurses should strengthen their understanding of presenteeism, which, in a sense, is not only harmful to their health but also to patient safety. Secondly, nurses should unite with each other, establish good interpersonal relationships, and develop a supportive team atmosphere. More importantly, managers should emphasize the harm presenteeism causes and give special populations more care and support.

## 5 Conclusion

Presenteeism is more severe among ICU nurses in public hospitals in Western China. Self-assessed health, perceived work stress, and having suffered from workplace violence in the past year are influential factors of presenteeism among ICU nurses, and ICU nurses’ occupational coping self-efficacy is closely related to presenteeism. Therefore, nursing managers should focus on nurses with moderate to high presenteeism and strengthen the prevention and intervention of presenteeism among nurses. In addition, this study has some limitations. First, this study is cross-sectional, which limits its ability to make causal arguments. Second, Due to potential bias in participants’ health self-assessments, there was a lack of exploration of other variables that may affect presenteeism and a focus on quantitative methods for analysis. Finally, the study is limited to certain hospital services, only ICU nurses in some public hospitals in western China were selected for the study, and there were some geographical limitations in the sample; the next step needs to carry out a multi-center, large-sample survey study to explore the characteristics and influencing factors of presenteeism among ICU nurses in hospitals in different regions.

## Data availability statement

The original contributions presented in the study are included in the article/Supplementary material, further inquiries can be directed to the corresponding authors.

## Ethics statement

The studies involving humans were approved by People’s Hospital of Deyang City (2021-04-056-K01). The studies were conducted in accordance with the local legislation and institutional requirements. The participants provided their written informed consent to participate in this study.

## Author contributions

JW: Conceptualization, Data curation, Formal analysis, Investigation, Methodology, Project administration, Software, Validation, Writing – original draft, Writing – review & editing. YL: Conceptualization, Data curation, Formal analysis, Investigation, Methodology, Software, Writing – original draft, Writing – review & editing. QL: Conceptualization, Data curation, Investigation, Methodology, Software, Writing – original draft, Writing – review & editing. JZ: Conceptualization, Data curation, Investigation, Methodology, Software, Writing – review & editing. ZL: Conceptualization, Data curation, Investigation, Methodology, Software, Writing – review & editing. XL: Conceptualization, Data curation, Formal analysis, Investigation, Methodology, Resources, Writing – review & editing. XZ: Conceptualization, Data curation, Funding acquisition, Investigation, Methodology, Resources, Supervision, Validation, Writing – review & editing. XR: Conceptualization, Funding acquisition, Investigation, Methodology, Project administration, Resources, Software, Validation, Writing – review & editing.
